# Antifungal Activity of *Bacillus velezensis* CE 100 against Anthracnose Disease (*Colletotrichum gloeosporioides*) and Growth Promotion of Walnut (*Juglans regia* L.) Trees

**DOI:** 10.3390/ijms221910438

**Published:** 2021-09-28

**Authors:** Vantha Choub, Henry B. Ajuna, Sang-Jae Won, Jae-Hyun Moon, Su-In Choi, Chaw Ei Htwe Maung, Chul-Woo Kim, Young Sang Ahn

**Affiliations:** 1 Department of Forest Resources, College of Agriculture and Life Sciences, Chonnam National University, Gwangju 61186, Korea; vanthachoub@gmail.com (V.C.); ajunahenry@mmu.ac.ug (H.B.A.); lazyno@naver.com (S.-J.W.); mjh132577@naver.com (J.-H.M.); Suin917@naver.com (S.-I.C.); 2 Division of Agricultural and Biological Chemistry, Institute of Environmentally Friendly Agriculture, College of Agriculture and Life Sciences, Chonnam National University, Gwangju 61186, Korea; chaweihtwemaung@gmail.com; 3 Division of Special-purpose Trees, National Institute of Forest Science, Suwon 16631, Korea; futuretree@korea.kr

**Keywords:** plant growth-promoting bacteria, lytic enzymes, auxin, root development, nutrient uptake, phytopathogenic fungi

## Abstract

Walnut anthracnose caused by *Colletotrichum gloeosporioides* is a deleterious disease that severely affects the production of walnut (*Juglans regia* L.). The aim of this study was to assess the antifungal and growth promotion activities of *Bacillus velezensis* CE 100 as an alternative to chemical use in walnut production. The crude enzyme from *B. velezensis* CE 100 exhibited chitinase, protease, and β-l,3-glucanase activity and degraded the cell wall of *C. gloeosporioides*, causing the inhibition of spore germination and mycelial growth by 99.3% and 33.6% at 100 µL/mL, respectively. The field application of *B. velezensis* CE 100 culture broth resulted in a 1.3-fold and 6.9-fold decrease in anthracnose disease severity compared to the conventional and control groups, respectively. Moreover, *B. velezensis* CE 100 produced indole-3-acetic acid (up to 1.4 µg/mL) and exhibited the potential for ammonium production and phosphate solubilization to enhance the availability of essential nutrients. Thus, field inoculation of *B. velezensis* CE 100 improved walnut root development, increased nutrient uptake, enhanced chlorophyll content, and consequently improved total biomass by 1.5-fold and 2.0-fold compared to the conventional and control groups, respectively. These results demonstrate that *B. velezensis* CE 100 is an effective biocontrol agent against anthracnose disease and a potential plant growth-promoting bacteria in walnut tree production.

## 1. Introduction

Walnut (*Juglans regia* L.) is the oldest cultivated fruit tree in Asia and has gained popularity around the world for edible oil production [1]. Walnut is considered a healthy food since it contains essential fatty acids and tocopherols that could reduce the risk of coronary heart diseases [2,3]. Besides the production of edible oil, walnuts are consumed as dry fruits and are valuable raw materials for confectionary, as well as bakery products, due to their health benefits [3,4]. Walnut trees are also among the most valuable sources of high-quality wood, for multiple uses, and products, such as veneers, furniture and interior designs, carving, gun stocks, and fore-ends [5]. In addition, the shells, kernels, seeds, barks, and leaves of walnut trees have been applied in the pharmaceutical and beauty industries due to their biochemical properties [3,6].

**Figure 1:**
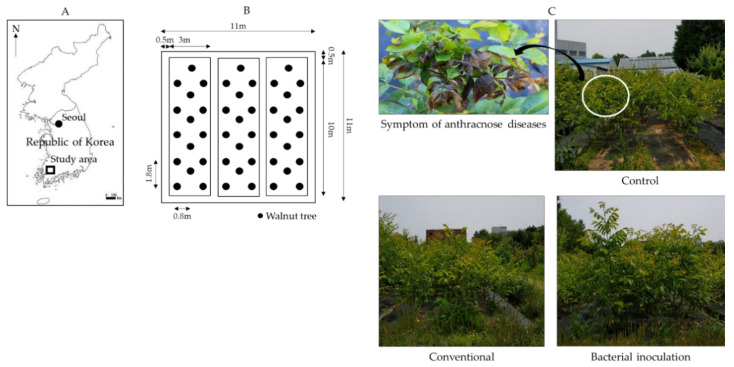
Study location of walnut orchard (**A**), arrangement of experimental site (**B**), and walnut tree showing symptoms of anthracnose disease caused by *Colletotrichum gloeosporioides* and walnut trees growing in the field experiment site (**C**).

However, walnut production can be adversely affected by diseases caused by phytopathogenic fungi, which reduce their yield and marketability by limiting tree growth and reducing the quality and edible size of nuts as well as their organoleptic properties [7,8]. Notably, anthracnose disease, caused by the *Colletotrichum gloeosporioides* species complex and the *C*. *acutatum* species complex, is among the most harmful and economically deleterious diseases in walnut production, especially in warm and humid conditions [9]. Walnut anthracnose is characterized by dieback and black spots on leaves and fruits that culminate into necrotic leaves with a reduced surface area for photosynthesis and fruit rot [8,9]. Similar symptoms were observed in this study (Figure 1C). The genus *Colletotrichum* is also an agent for anthracnose diseases in various other hosts plants, including tea (*Camellia sinensis* L. Kuntze), tulip tree (*Liriodendron tulipifera* Linnaeus), mangos (*Mangifera indica* Linn.), and avocados (*Persea americana* Mill) [10,11,12]. *C. gloeosporioides* species are hemibiotrophic pathogens with cross-infection potential as well as genetic and pathogenic heterogeneity, making them more versatile, infectious, and thus economically important [9,11,13]. As the fungus sporulates, masses of orange conidia are observed around dry necrotic lesions, which later disperse to infect other plant parts, causing twig dieback, severe loss of yield, and a reduced fruit quality [8,14,15]. Due to their multiple host ranges, there are numerous sources of *C. gloeosporioides* infection, including dead contaminated leaves, defoliated branch terminals, dead panicles, fruits, and bracts in close proximity, during favorable conditions [13,15,16]. The conidia of *C. gloeosporioides* can spread to susceptible hosts by several means, including irrigation, light rain, heavy dew, and fog [13,16].

Conventional fungicides, such as tebuconazole, difenoconazole, carbendazim, benomyl, thiabendazole, and prochloraz, have been used to control phytopathogens that cause anthracnose disease in orchards [12,15]. However, the continuous use of agricultural chemicals is associated with health and environmental risks, including spray drift hazards that adversely affect the environment [17], fungicide resistance [18], and detrimental effects on the health of the farmer and consumers [17,19]. Despite the existence of policies and regulatory authorities regarding the usage of pesticides in various countries, the lack of adherence and compliance due to various social–economic and political reasons pose a significant risk. For instance, in South Korea, dithiocarbamate (mancozeb) fungicides were classified as number one among the class of endocrine-disrupting substances (CAT1) based on the evidence of endocrine-disrupting activity in an intact organism, but they remained the second most used chemical pesticide according to a 2014 report [20]. Therefore, there is a growing demand for crop production strategies that can reduce the use of agrochemicals, for instance, the use of microbial biocontrol agents as an alternative to fungicides. Environmentally friendly practices, such as biocontrol agents, are also important for promoting plant growth [21,22,23]. Thus, the role of active metabolites derived from plant growth-promoting bacteria (PGPB), such as the *Bacillus* species, as potential alternatives to chemical fungicides cannot be underestimated [24,25].

Many researchers have discovered new microbial candidates and agricultural engineering techniques that use bio-resources, such as PGPB, as biocontrol agents as well as biofertilizers for enhancing plant growth and production [25,26]. Biocontrol agents can colonize the plant rhizosphere, and secrete metabolites that control phytopathogens [25], and act as bio-fertilizers to enhance plant growth [26]. *Bacillus* spp., such as *B. subtilis*, *B. licheniformis*, *B. amyloliquefaciens*, and *B. velezensis,* are among the PGPB candidates that have been reported to have potential applications as biocontrol agents and plant growth-promoting bacteria because of their prominent traits [21,22,23,24,25]. PGPB have different biocontrol mechanisms against phytopathogenic fungi, including the production of secondary metabolites, such as antibiotics and lipopeptides [25,27]. For instance, a previous study found that *B. velezensis* CE 100 can produce cyclic tetrapeptide with antifungal activities against the mycelial growth and spore germination of plant pathogenic fungi, such as *C. gloeosporioides* [27]. However, such compounds are often produced at very low concentrations in the bacterial culture broth. Therefore, they are not likely to be the main mechanism for the antifungal activity of *B. velezensis* CE 100 in the field [22,27]. PGPB also control phytopathogenic fungi through the secretion of cell wall-degrading enzymes, such as chitinase, protease, and β-l,3-glucanase [21,22,28]. These cell wall-degrading enzymes secreted by antagonistic bacteria can degrade the cell wall of phytopathogenic fungi, which causes the inhibition of mycelial growth and spore germination [28,29,30]. Fungal cell wall-degrading enzymes have also been exploited through biotechnological techniques at industrial scale due to their antifungal activities [31]. Therefore, the production of lytic enzymes, such as chitinase, protease, and β-l,3-glucanase, by antagonistic bacteria, such as *B. velezensis*, is an essential trait for their biocontrol potential against phytopathogenic fungi.

Besides the biocontrol of phytopathogens, PGPB can be applied as biofertilizers to enhance plant growth and yield in agriculture and forestry [26,32]. PGPB can secrete phytohormones, such as indole-3-acetic acid (IAA) auxin, which promotes root hair and lateral root growth in plants [33,34]. This increases the root surface area for nutrient uptake [33,34]. The enhanced nutrient absorption increases the photosynthetic rate, leading to improved plant growth and productivity [32,34]. For instance, increased nitrogen absorption improves chlorophyll content, which ultimately enhances the photosynthetic rate and biomass production of plants treated with PGPB [35,36]. Moreover, some PGPB, such as *B. velezensis*, play an important role in nutrient cycling in soil and can thus enhance plant productivity by increasing the availability and absorption of nutrients [35,37]. Previous studies have demonstrated the potential of several *Bacillus* spp. to control fungal diseases, such as anthracnose, and promote the growth of various plants [37,38]. However, there is limited knowledge about the effective application of *Bacillus* spp. to simultaneously control anthracnose disease and enhance the growth of walnut trees at the field scale. Thus, the objective of this study was to assess the potential of *B. velezensis* CE 100 to simultaneously control walnut anthracnose caused by *C. gloeosporioides* and promote the growth of walnut trees at the field scale. Inoculation of *B. velezensis* CE 100 effectively reduced anthracnose severity through the production of fungal cell wall-degrading enzymes, such as chitinase, protease, and β-l,3-glucanase. *B. velezensis* CE 100 also promoted the growth of walnut trees through the secretion of IAA, ammonium production, and phosphate solubilization. Therefore, *B. velezensis* CE 100 could potentially be applied as a biocontrol agent as well as a biofertilizer in walnut production as an alternative to chemical use in orchard management. 

## 2. Results

### 2.1. Biocontrol of Walnut Anthracnose Disease Using Bacillus Velezensis CE 100

#### 2.1.1. Changes in Cell Growth of Bacillus velezensis CE 100

**Figure 2:**
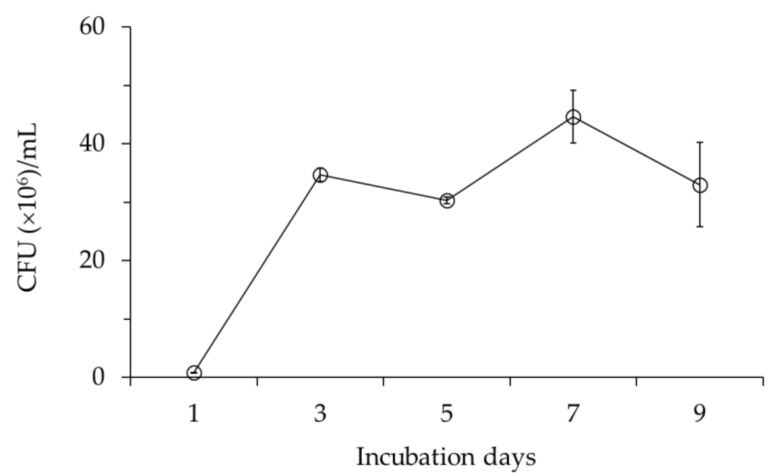
Cell growth of *Bacillus velezensis* CE 100 during incubation period. Error bars represent the standard deviation (number of values in the sample (*n*) = 3).

The cell growth of *B. velezensis* CE 100 increased to a peak value of 4.4 × 10^7^ colony-forming units (CFU)/mL after 7 days of incubation and then declined (Figure 2). 

#### 2.1.2. Chitinase, Protease, and β-l,3-Glucanase Activity in the Culture Broth and Crude Enzyme of Bacillus Velezensis CE 100

**Figure 3:**
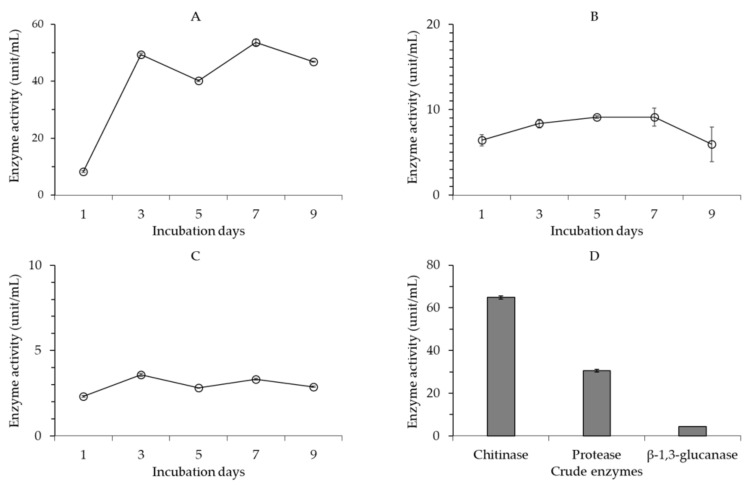
Changes in the activity of lytic enzymes from *Bacillus velezensis* CE 100 culture broth during the incubation period; chitinase activity (**A**), protease activity (**B**), β-l,3-glucanase activity (**C**), and activity of lytic enzymes in the crude enzyme solution from *B. velezensis* CE 100 (**D**). Error bars represent the standard deviation (*n* = 3).

Chitinase activity in the cell-free culture broth of *B. velezensis* CE 100 increased in two phases, with an initial increase 3 days after inoculation followed by a slight decline 5 days after inoculation. Then, chitinase activity increased again to a maximum of 53.7 unit/mL at 7 days before the final decline (Figure 3A). The pattern of chitinase production by *B. velezensis* CE 100 was consistent with bacterial cell growth. Protease activity in the cell-free culture broth of *B. velezensis* CE 100 increased steadily (independent of the cell growth pattern) to a maximum of 9.1 unit/mL 7 days after inoculation and then significantly declined (Figure 3B). The pattern of β-l,3-glucanase activity in the cell-free culture broth of *B. velezensis* CE 100 was relatively consistent with the cell growth pattern (Figure 3C). The highest β-l,3-glucanase activity of 3.6 unit/mL was observed 3 days after inoculation. The activity of β-l,3-glucanase declined for 5 days before the second increase to 3.3 unit/mL at 7 days. The lytic enzyme activity of chitinase, protease, and β-l,3-glucanase in the cell-free culture broth of *B. velezensis* CE 100 was generally the highest 7 days after inoculation, which is consistent with the highest cell growth (Figure 3A–C). The maximum enzyme activity for chitinase exhibited by the cell-free culture broth of *B. velezensis* CE 100 was 5.9-fold and 14.9-fold higher compared to the maximum enzyme activity for protease and β-l,3-glucanase, respectively.

The activity of lytic enzymes in the crude enzyme obtained from the 7-day cell-free culture broth of *B. velezensis* CE 100 indicates that the chitinase enzyme exhibited the highest activity of 64.9 unit/mL (Figure 3D). The activity of protease and β-l,3-glucanase in the crude enzyme was 30.5 unit/mL and 4.4 unit/mL, which is 2.1-fold and 14.7-fold lower compared to chitinase activity. 

#### 2.1.3. Activity of Crude Enzyme from Bacillus velezensis CE 100 against Spore Germination and Mycelial Growth of Colletotrichum Gloeosporioides

**Figure 4:**
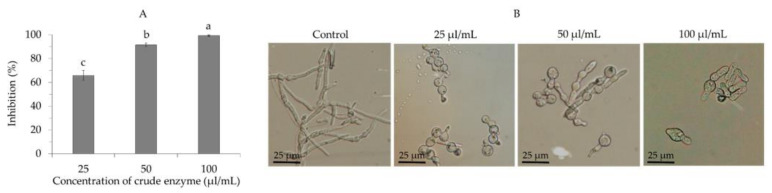
Antifungal activity of different concentrations of crude enzyme from *Bacillus velezensis* CE 100 against spore germination of *Colletotrichum gloeosporioides*; percentage inhibition of spore germination (**A**) and spore germination and germ tube elongation (**B**). Error bars represent the standard deviation (*n* = 3). Means with different superscripts are significantly different (*p* < 0.001).

The crude enzyme obtained from the 7-day cell-free broth culture of *B. velezensis* CE 100 exhibited a strong efficacy against the germination of *C. gloeosporioides* spores. Treatment with the crude enzyme at concentrations of 100 µL/mL, 50 µL/mL, and 25 µL/mL caused spore germination inhibition by 99.3%, 91.6%, and 66.0%, respectively (Figure 4A). Treatment with the crude enzyme at each concentration significantly reduced the ability of germ tube elongation in the case of germinated spores compared to normal germ tube elongation observed in the control group (Figure 4B). 

**Figure 5:**
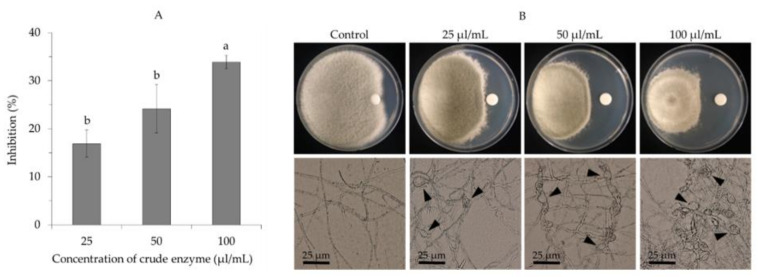
Antifungal activity of different concentrations of crude enzyme from *Bacillus velezensis* CE 100 against mycelial growth of *Colletotrichum gloeosporioides*; percentage inhibition of mycelial growth (**A**), and hyphae morphology (**B**). Arrows indicate deformed mycelia after treatment with crude enzyme. Error bars represent the standard deviation (*n* = 3). Means with different superscripts are significantly different (*p* = 0.003).

Moreover, the crude enzyme from *B. velezensis* CE 100 inhibited the mycelial growth of *C. gloeosporioides* (Figure 5A). The highest mycelial inhibition of 33.6% was observed at a concentration of 100 µL/mL of the crude enzyme, while the lowest inhibition rate of 16.8% was recorded at 25 µL/mL of the crude enzyme. All treatment concentrations (25 µL/mL, 50 µL/mL, and 100 µL/mL) of the crude enzyme caused an abnormal hyphae morphology in the mycelia of *C. gloeosporioides* (Figure 5). When treated with the crude enzyme, the hyphae exhibited an uneven distribution of the cytoplasm, cell wall lysis, and swelling (shown by arrows), while the hyphae in the control group showed normal growth without deformation (Figure 5B).

#### 2.1.4. Biocontrol of Walnut Anthracnose Disease with Bacillus Velezensis CE 100 Culture Broth under Field Conditions

**Figure 6:**
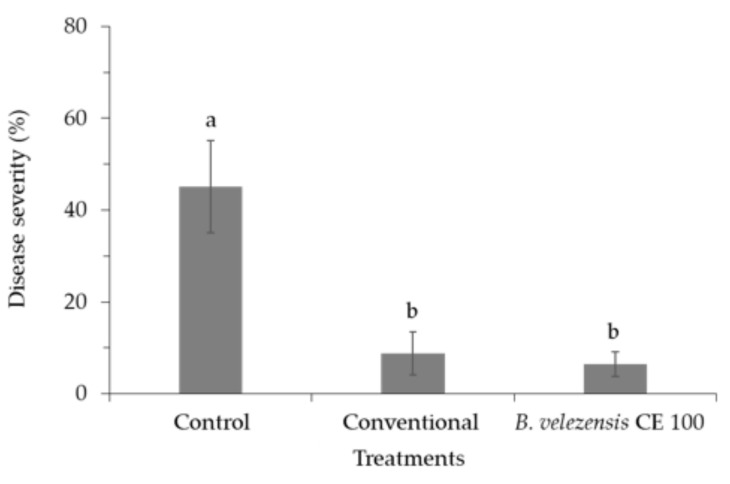
The severity of anthracnose disease on the leaves of walnut trees under different treatments. Error bars represent the standard deviation (*n* = 12). Means with different superscripts are significantly different (*p* < 0.001).

Inoculation of *B. velezensis* CE 100 culture broth on walnut trees resulted in significantly lower anthracnose disease severity (leaf necrosis) compared to the control group (Figure 6). Walnut trees inoculated with *B. velezensis* CE 100 showed only 6.5% disease severity compared to 8.7% in the conventional treatment and 45.1% in the control group. Inoculation with *B. velezensis* CE 100 culture broth reduced anthracnose disease severity by 1.3-fold and 6.9-fold compared to the conventional treatment and the control groups, respectively. The leaves of the walnut trees in the control group showed severe brown spots of necrotic lesions due to *C. gloeosporioides* infection. 

### 2.2. Plant Growth Promotion Activity of Bacillus velezensis CE 100 

#### 2.2.1. Indole-3-Acetic Acid (IAA) Production 

**Figure 7:**
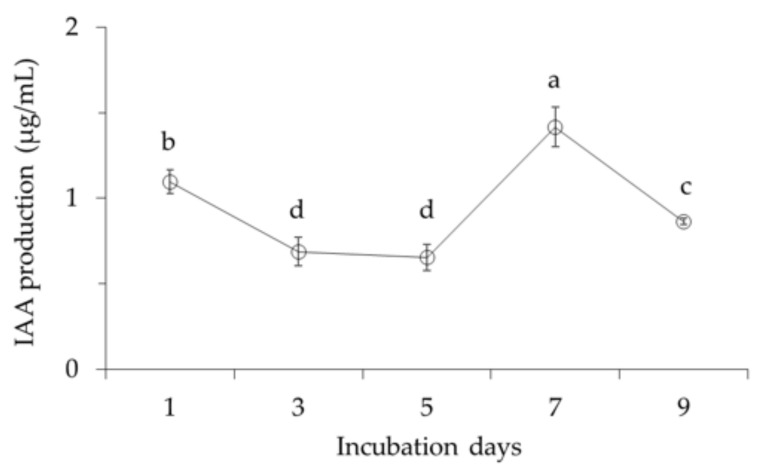
IAA production activity of *Bacillus velezensis* CE 100. Error bars represent the standard deviation (*n* = 3). Means with different superscripts are significantly different (*p* < 0.001).

The highest IAA production was 1.4 µg/mL at 7 days of incubation (Figure 7). After 7 days, a slight decrease in IAA production was then observed, which also corresponds with the maximum cell growth.

#### 2.2.2. Ammonium Production and Phosphate Solubilization

**Figure 8:**
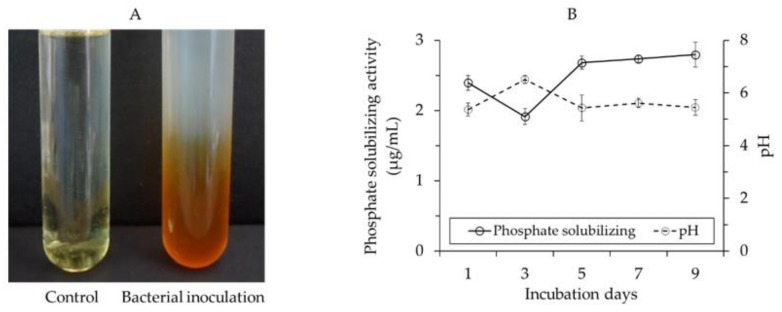
Ammonium production activity (**A**), phosphate-solubilizing activity and pH changes (**B**) of *Bacillus velezensis* CE 100. Error bars represent standard deviation (*n* = 3).

When grown in peptone broth for 2 days, *B. velezensis* CE 100 exhibited ammonium production activity as evidenced by a change from a faint yellow to a dark brown color in Nessler’s reagent (Figure 8A). The positive reaction in Nessler’s reagent indicates the production of ammonium by *B. velezensis* CE 100, which is a precursor for nitrate in soil.

In addition, *B. velezensis* CE 100 showed phosphate-solubilizing activity as indicated by the solubilization of tricalcium phosphate (TCP) in Pikovskaya’s (PVK) medium with an associated drop in pH (Figure 7B). The bacterial culture broth of *B. velezensis* CE 100 incubated in PVK medium showed a drop in pH from 6.5 at 3 days to 5.4 at 9 days. On the other hand, the soluble phosphate concentration in the PVK medium increased from 1.9 µg/mL at 3 days to 2.8 µg/mL at 9 days of incubation (Figure 8B).

#### 2.2.3. Growth Promotion and Biomass Production of Walnut Trees

The average chlorophyll content (single-photon avalanche diode (SPAD) units) in walnut trees inoculated with *B. velezensis* CE 100 was 44.6 units compared to 39.0 units and 33.2 units in the conventional and control groups, respectively (Table 1). This indicates that inoculation with *B. velezensis* CE 100 increased the chlorophyll content in the leaves of walnut trees by 1.1-fold and 1.3-fold compared to the conventional treatment and control groups, respectively.

In addition, walnut trees inoculated with *B. velezensis* CE 100 had significantly higher shoot length, root collar diameter, and biomass compared to the conventional treatment and control groups (Table 1). The average shoot lengths of the walnut trees were 227.6 cm, 187.0 cm, and 184.6 cm in the *B. velezensis* CE 100 inoculation, conventional treatment, and control groups, respectively. Inoculation with *B. velezensis* CE 100 culture broth increased the average shoot length by 1.2-fold compared to both the conventional treatment and control groups. In addition, the average root collar diameters of the walnut trees were 57.2 mm, 42.4 mm, and 44.3 mm for the *B. velezensis* CE 100 inoculation, conventional treatment, and control groups, respectively (Table 1). Thus, inoculation with *B. velezensis* CE 100 culture broth increased root collar diameter by 1.4-fold and 1.3-fold compared to the conventional treatment and control groups, respectively. Moreover, an average biomass of 1193.3 g, 795.5 g, and 613.5 g was recorded for the *B. velezensis* CE 100 inoculation, conventional treatment, and control groups, respectively (Table 1). Thus, inoculation with *B. velezensis* CE 100 increased biomass in walnut trees by 1.5-fold and 2.0-fold compared to the conventional treatment and control groups, respectively.

#### 2.2.4. Nutrient Contents of Walnut Trees 

Inoculation with *B. velezensis* CE 100 enhanced nutrient uptake in walnut trees compared to the conventional treatment and control groups (Table 2). The average total nitrogen content in walnut trees inoculated with *B. velezensis* CE 100 was 14.7 g/plant compared to 13.1 g/plant and 9.9 g/plant in the conventional treatment and control groups, respectively. Thus, inoculation with *B. velezensis* CE 100 increased the total nitrogen content in walnut trees by 1.1-fold and 1.5-fold compared to the conventional treatment and control groups, respectively (Table 2). Similarly, the total phosphorus content in walnut trees in the *B. velezensis* CE 100 inoculation, conventional treatment, and control groups were 2.2 g/plant, 1.5 g/plant, and 1.4 g/plant, respectively. Thus, the application of *B. velezensis* CE 100 increased the total phosphorus content by 1.5-fold and 1.6-fold compared to the conventional treatment and control groups, respectively (Table 2). 

## 3. Discussion

### 3.1. Antagonistic Activity of Bacillus velezensis CE 100 against Colletotrichum gloeosporioides in Walnut Trees 

In this study, *B. velezensis* CE 100 showed antifungal activity against *C. gloeosporioides*, the causal agent of anthracnose disease in walnut trees (Figure 4 and Figure 5). Previously, Choub et al. [27] indicated that *B. velezensis* CE 100 can produce antifungal cyclic tetrapeptides, which caused 100% spore germination inhibition and 18.8% mycelial growth inhibition against *C. gloeosporioides* at a concentration of 1000 µg/mL. However, cyclic tetrapeptide could neither inhibit mycelial growth at 500 µg/mL nor prevent spore germination at 250 µg/mL. Moreover, only 0.5 mg of cyclic tetrapeptide could be obtained from 1.5 L of *B. velezensis* CE 100 culture broth. Therefore, the low concentration of cyclic tetrapeptide from *B. velezensis* CE 100 culture broth is not likely to possess antifungal activity under field conditions. On the other hand, lytic enzymes, such as β-1,3-glucanase and protease produced by *B. velezensis* CE 100, were previously reported to cause antifungal activity against several phytopathogenic oomycetes (*Phytophthora* spp.) in forest seedlings [39].

Phytopathogenic fungal virulence and environmental adaptability are to a great extent influenced by the properties of the cell wall, which is mainly composed of cross-linked chitin, modified glycoprotein, and glucan polymers [30,31,40]. Fungal cell wall chitin (composed of β-(1,4)-linked *N*-acetyl-D-glucosamine) and glucans are synthesized in the plasma membrane and extruded into the cell wall space, while the glycoproteins are synthesized by endoplasmic reticulum-bound ribosomes and are modified (glycosylation) by cross-linking the proteins into the cell wall matrix to provide cell wall integrity [40]. Thus, lytic enzymes, such as chitinase, protease, and β-1,3-glucanase, can effectively hydrolyze chitin, glucan, and glycoprotein polymers, which are the most essential structural components in the cell wall matrix of phytopathogenic fungi [30,31,40]. Chitinase, protease, and β-1,3-glucanase enzymes degrade the hyphae of phytopathogenic fungi, causing cell wall lysis and deformation, which consequently inhibits mycelial growth [29,30,31]. Moreover, these lytic enzymes also contribute to the biocontrol activity of antagonistic bacteria by inhibiting the germination and adhesion of phytopathogenic fungal spores, as well as appressoria formation to prevent plant infection [28]. In this study, *B. velezensis* CE 100 exhibited chitinase, protease, and β-1,3-glucanase activity during growth (Figure 3). The crude enzyme from *B. velezensis* CE 100 exhibited a strong antifungal activity against *C. gloeosporioides*. Specifically, treatment with 100 µL/mL of the crude enzyme inhibited the spore germination and mycelium growth of *C. gloeosporioides* by 99.3% and 33.6%, respectively (Figure 4A and Figure 5A). The inhibitory effect of the crude enzyme against *C. gloeosporioides* was characterized by the deformation of the cellular structures (Figure 5B), which indicates the potential lysis of the cross-linked polymers of chitin, glucans, and glycoproteins in the fungal cell wall, by chitinase, protease, and β-1,3-glucanase enzymes produced by *B. velezensis* CE 100 [29,30,31,40]. Similarly, the inhibition of the germ tube elongation in germinated spores could be attributed to the activity of the lytic enzymes produced by *B. velezensis* CE 100 [28,38,40]. Hyphae cell wall degradation, the inhibition of spore germination, germ tube elongation, and spore adhesion by lytic enzymes (such as chitinase, protease, and β-1,3-glucanase) not only reduce the phytopathogenic fungal density but also prevent appressorium formation and, thus, reduce the infection rate of *C. gloeosporioides* on walnut trees inoculated with *B. velezensis* CE 100 [28,29,30,31]. These observations are consistent with the study of Huang et al. [38], where the metabolite-produced *B. velezensis* strain HYEB5-6 inhibited conidial germination, germ tube growth, and appressorium formation of phytopathogenic *C. gloeosporioides*. However, studies on the field application of PGPB in the management of tree diseases are still limited. Our results demonstrate that field application of *B. velezensis* CE 100 culture broth reduced anthracnose disease severity by 85.6% compared to the control (Figure 6). On the other hand, the application of the conventional treatment reduced anthracnose disease severity only by 80.6% compared to the control, which was lower than the effect of *B. velezensis* CE 100 inoculation (Figure 6). *B. velezensis* strains have been previously reported to exhibit effective biocontrol activity against some plant fungal pathogens in the field. For instance, Castro et al. [41] found that *B. velezensis* XT1, when applied directly onto young olive trees, effectively suppressed *Verticillium* wilt incidence rates and reduced the disease severity by 50–60%. Their findings are consistent with the results of this study, which demonstrate that *B. velezensis* CE 100 could be applied in the field as an effective biocontrol agent against *C. gloeosporioides*, the causal agent of anthracnose disease in walnut trees.

### 3.2. Growth Promotion of Walnut Trees by Bacillus velezensis CE 100 

In this study, *B. velezensis* CE 100 exhibited ammonia production activity (Figure 8A), and, thus, its inoculation in the soil could improve the total nitrogen content. In the soil, ammonium is a percussor for nitrite, which is then naturally converted to nitrate (the most absorbable form of soil nitrogen) by means of nitrifying bacteria involved in the nitrogen cycle [35,42]. Nitrogen is a central component of plant protein and chlorophyll pigment, and its uptake can directly enhance the photosynthetic rate [36,43]. In addition, *B. velezensis* CE 100 exhibited phosphate-solubilizing activity (Figure 8B). This indicates that *B. velezensis* CE 100 has the potential to increase phosphate availability in the rhizosphere by changing insoluble phosphate into a soluble state (P_3_O_5_^−^) for plant uptake [35,44]. In plants, phosphate is an essential nutrient that is specifically responsible for root development. Therefore, the availability of phosphate in the soil can indirectly affect the uptake of other nutrients with a low diffusion coefficient by enhancing the root architecture [35,45]. Specifically, when the rate of nutrient uptake is affected by root density, the role of phosphate in the soil becomes vital in the absorption of other essential nutrients. Moreover, phosphorus also plays a key role in plant physiological processes, including photosynthesis [35,45,46].

In this study, *B. velezensis* CE 100 increased the total nitrogen and total phosphorus uptake in the walnut trees (Table 2). This consequently improved the chlorophyll content in walnut trees by 1.1-fold and 1.3-fold compared to the conventional treatment and control groups, respectively (Table 1). These results demonstrate that *B. velezensis* CE 100 improved nutrient availability and nutrient uptake and enhanced the photosynthetic rate, which consequently increased the growth and biomass production of walnut trees (Table 1).

Moreover, *B. velezensis* CE 100 produced indole-3-acetic acid (IAA) auxin (Figure 7). Auxins can directly initiate the division of lateral root founder cells in the pericycle tissue. They also initiate the emergence and development of lateral root primordia indirectly from adjacent root cortical tissues [47]. Auxins from emerging lateral roots can also cause a local inductive signal that re-programs adjacent cells to promote root hair development [47,48]. Therefore, auxin production by PGPB, such as *B. velezensis* CE 100, can increase the surface area for nutrient uptake by promoting the growth of root hairs and lateral roots in plants [45]. This phenomenon ultimately enhanced walnut tree growth (Table 1). The results of this study are consistent with the previous studies that demonstrated an enhanced growth and productivity of different orchard trees, including olive trees, by PGPB [41]. These results demonstrate that *B. velezensis* CE 100 could potentially be applied in the field as a biofertilizer for improving tree growth in orchards and forests.

## 4. Materials and Methods

### 4.1. Antagonistic Bacteria and Phytopathogenic Fungi Preparation

Antagonistic bacterium *B. velezensis* CE 100 was previously isolated from the pot soil of a tomato plant [49,50], and the stock culture (10^7^ CFU/mL) was mixed with 50% glycerin and maintained at −80 °C for use in further experiments.

The fungal pathogen used in this study was isolated from diseased leaves of walnut trees in the forest resource field site (Figure 1) at Chonnam National University, Gwangju, Korea (approximately 35°17′ N latitude, 126°90′ E longitude). The surfaces of the leaves were disinfected with 0.5% sodium hypochlorite solution for 2 min, then washed thrice in sterile distilled water, and dried on sterilized cotton wool. The leaves were cut into small sections (1 × 1 cm^2^) with a sterilized surgical blade and inoculated at the center of potato dextrose agar (PDA) plates pre-treated with streptomycin sulfate (50 μg/L) to limit bacterial growth. After 10 days of incubation at 25 °C, fungi hyphal tips were sub-cultured twice on fresh PDA plates without streptomycin sulfate. Four distinct fungal isolates were obtained based on the morphological characteristics of the mycelia and conidia. 

Only one isolate was confirmed for pathogenicity of walnut anthracnose based on Koch’s postulates technique, as evidenced by the ability to cause infection (black spots) on the leaves of healthy walnut trees. The isolate was subjected to 18S RNA gene sequence at Macrogen Inc. laboratory (Seoul, Korea). Phylogenetic analysis was conducted by comparing the obtained gene sequences with existing data previously deposited in GenBank (https://blast.ncbi.nlm.nih.gov/Blast.cgi, accessed on 25 September 2019) using the MEGA 7 software system. The analysis was based on the maximum likelihood method, and the phytopathogenic fungal strain responsible for walnut anthracnose was identified as *C. gloeosporioides*. Then, *C. gloeosporioides* was sub-cultured on PDA medium at 25 °C for 7 days for further experiments.

### 4.2. Cell Growth and Activity of Lytic Enzymes produced by Bacillus velezensis CE 100

To determine cell growth and lytic enzyme activity, a colony of *B. velezensis* CE 100 was inoculated into tryptic soy broth (TSB) and incubated at 30 °C for 3 days at 130 rpm in a shaking incubator. Then, 1 mL/L of *B. velezensis* CE 100 culture broth was inoculated into pink broth (PB) medium containing colloidal chitin 1%, pink fertilizer 3 g, KH_2_PO_4_ 0.2 g, MgSO_4_ 0.2 g, CaCO_3_ 0.1 g, NaCl 0.1 g, sucrose 3 g, and water 1 L. The bacterial culture broth samples were taken on 1, 3, 5, 7, and 9 days during the incubation period to analyze cell growth and lytic enzyme activity. Cell growth was determined by cell counting (CFU/mL) on tryptic soy agar (TSA) media. To determine the activity of lytic enzymes, samples were then centrifuged at 12,000 rpm for 15 min, and the supernatants were used to determine chitinase, protease, and β-1,3-glucanase activity.

Chitinase production was determined based on the degradation of chitin substrate using the method described previously [51]. After centrifugation, 50 µL of the supernatant of each sample was mixed with 500 µL of 0.5% colloidal chitin and 450 µL of sodium acetate buffer (pH 5.0) and incubated at 37 °C for 1 h. The reaction was stopped by adding 200 µL of 1N NaOH. After vortexing, the precipitant was removed by centrifugation at 12,000 rpm for 7 min. Then, 750 µL of the supernatant was mixed with 250 µL of water and 1000 µL of Schales reagent. Then, the mixture was boiled for 15 min, and the absorbance was measured at 420 nm using a UV spectrophotometer (Shimadzu UV-1700, Kyoto, Japan). The chitinase activity was calculated from the standard curve (unit/mL). One unit of chitinase activity was defined as the reducing activity that released 1 µmol of N-acetyl-glucosamine/hour at 37 °C.

Protease activity was determined as previously described [39]. Briefly, 100 mM of tris buffer containing 2 mM CaCl_2_ and 1% casein was prepared and adjusted to pH 8.0. A reaction mixture containing 50 µL of cell-free bacterial culture broth and 950 µL of tris buffer was incubated at 60 °C for 15 min. Then, 500 µL of 20% trichloroacetic acid was added to terminate the reaction. The mixture was centrifuged at 13,000 rpm for 15 min, and the absorbance of the supernatant containing acid-soluble proteins was measured at 280 nm using a UV spectrophotometer. One unit of protease activity was defined as the amount of enzyme that liberated 1 µg of tyrosine per min.

β-l,3-glucanase activity was determined by measuring the glucose released from laminarin during a glucose oxidase reaction as previously described [39]. Briefly, 0.4 mL of 50 mM sodium acetate (pH 5.0), 50 µL of 1% laminarin solution, and 50 µL of cell-free *B. velezensis* culture broth were reacted in a water bath at 37°C for 1 h. Then, 1.5 mL of dinitrosalicylic acid solution was added, and the solution was boiled for 5 min to stop the reaction. For the blank samples, water was added to the reaction instead of the bacterial culture to serve as a control. The concentration of reducing sugars was measured at 550 nm using a UV spectrophotometer. One unit of β-1,3-glucanase activity was defined as the amount of enzyme that catalyzed the release of 1 µmol of glucose/mg of protein/hour. The protein concentration of the samples was determined in triplicate using the Bradford method [49]. Briefly, 100 µL of cell-free *B. velezensis* culture broth was added to 1 mL of Bradford reagent, Coomassie blue G-250 (Sigma-Aldrich, Darmstadt, Germany), and incubated at room temperature for 15 min. The resultant blue color was measured at 595 nm using a UV spectrophotometer. The standard curve was plotted using seven serial dilutions (0.25 mg/mL to 2 mg/mL) of standard bovine serum albumen, BSA (Sigma-Aldrich, Darmstadt, Germany). The analysis was repeated three times to obtain consistent results.

### 4.3. Preparation of Crude Enzyme from Bacillus velezensis CE 100

For crude enzyme preparation, *B. velezensis* CE 100 was grown in PB medium and incubated at 30 °C for 7 days with shaking at 130 rpm. After the incubation period, *B. velezensis* CE 100 culture broth was centrifuged at 6000 rpm for 1 h at 4 °C and then filtered through four layers of filter paper (Whatman No.6, Whatman International Ltd., Maidstone, UK). *B. velezensis* CE 100 cell-free culture broth was extracted by precipitation using ammonium sulfate as previously described [51]. The supernatant was collected and precipitated with ammonium sulfate saturated solution dropwise until 80% saturation. While gently stirring, the mixture was allowed to stand at 4 °C overnight. The precipitate containing crude enzyme was harvested by centrifugation at 6000 rpm for 1 h at 4 °C. The pellet was dissolved in a minimal amount of 20 mM Tris-HCl buffer (pH 8.2). The crude enzyme solution was then dialyzed extensively at 4 °C against 20 mM Tris-HCl buffer (pH 8.2). Polyethylene glycol was used to remove the Tris-buffer from the dialysis tubing. The crude enzyme was dissolved in a small amount of 20 mM Tris-HCl buffer (pH 8.2). The chitinase, protease, and β-1,3-glucanase activity of the crude enzyme was determined as described above based on the degradation of chitin, casein, and laminarin substrates, respectively. The protein concentration of the crude enzyme was determined using the Bradford method [49] as described above. The obtained crude enzyme was then kept at −80 °C for further use.

### 4.4. Antifungal Activity of Crude Enzyme on Spore Germination and Mycelial Growth of Colletotrichum gloeosporioides

To determine the inhibitory effect of crude enzyme from *B. velezensis* CE 100 on spore germination, the spore suspension was prepared from the 7-day culture of *C. gloeosporioides* grown on PDA medium at 28 °C. The fungal culture plates were then flooded with 10 mL of sterile distilled water followed by gentle scrubbing with a sterilized spatula. The suspension was then filtered through four layers of sterilized cheesecloth. The number of spores in the suspension was counted using a hemocytometer and adjusted to 10^6^ spores/mL. Then, crude enzyme was applied at different concentrations (25 µL/mL, 50 µL/mL, and 100 µL/mL) in 20 mM Tris-HCl buffer (pH 8.2). For the control group, the same spore concentration was used, and crude enzyme was replaced with an equal volume of 20 mM Tris-HCl buffer (pH 8.2). Each experiment was performed in three replications in Eppendorf tubes containing 250 µL of sterile 4 × PDA (9.6 g/100 mL), 100 µL of spore suspension, and crude enzyme for each respective concentration. The final volume was adjusted to 1 mL with sterile distilled water and incubated at 25 °C for 10 h. Then, 10 µL of the solution was used to count the number of germinated spores using a hemocytometer. A total of 100 spores from each Eppendorf tube were examined using a light microscope (Olympus BX41, Tokyo, Japan) at 200× magnification. The spore germination inhibition (%) was calculated using the following formula: spore germination inhibition (%) = (GC − GT/GC) × 100, where GC is the total number of germinated spores in the control group, and GT is the total number of germinated spores in the treatment group.

The inhibitory effect of the crude enzyme from *B. velezensis* CE 100 on the mycelial growth of *C. gloeosporioides* was determined using a paper disc assay (disc diffusion method) as previously described [27]. Crude enzyme was prepared in 20 mM Tris-HCl buffer (pH 8.2) at different concentrations (25 µL/mL, 50 µL/mL, and 100 µL/mL). Then, 25 µL from each concentration of crude enzyme was loaded onto the paper disc. The paper discs were placed on one the side of a PDA plate. The same PDA plate was inoculated with a mycelial plug (5 mm in diameter) of *C. gloeosporioides* at a 4 cm distance from the paper disc. The paper discs for the control group were loaded with 25 µL of 20 mM Tris-HCl buffer (pH 8.2). Each treatment was prepared in three replicates, and the plates were incubated at 25 °C for 7 days. Mycelial growth inhibition was calculated with the following equation: mycelial growth inhibition (%) = (M − m)/M × 100, where M is the radical mycelial growth in the control plate, and m is the radical of mycelial growth in the dual dish. To examine the effect of each treatment on the mycelial morphology of *C. gloeosporioides*, small pieces of mycelial growth on the border between the paper disc and fungal growth were examined for hyphae deformation. The mycelia were placed on a glass slide with a drop of water, covered with glass coverslips, and examined under a light microscopic at 200× magnification.

### 4.5. Plant Growth Promotion of Bacillus velezensis CE 100

#### 4.5.1. Indole-3-Acetic Acid (IAA) Activity

IAA production by *B. velezensis* CE 100 was determined on PB medium amended with L-tryptophan (0.1 g/L) as previously described [39]. The cell culture broth (1 mL/L) of *B. velezensis* CE 100 (10^7^ CFU/mL) was inoculated in the sterilized medium and incubated at 30 °C for 3 days in a shaking incubator at 130 rpm. During the incubation, 5 mL of cell culture broth was sampled on 1, 3, 5, 7, and 9 days. The cell culture broth was centrifuged at 12,000 rpm for 15 min. To test for IAA activity, 1 mL of supernatant was mixed with 2 mL Salkowski reagent (50 mL of 35% perchloride acid and 1 mL of 0.5 M FeCl_3_), and then two drops of phosphoric acid were added. The test tubes containing the treatments were incubated at room temperature in the dark condition for 25 min. For the blank sample (control), the bacteria culture was replaced with water. The development of a pink coloration indicated the production of IAA activity. The absorbance of the samples was measured at 530 nm using a UV spectrometer, and IAA production was calculated based on the equation obtained from the standard curve.

#### 4.5.2. Ammonium Production and Phosphate-Solubilizing Activity

The ammonium production activity of *B. velezensis* CE 100 was tested using the method described by Ahmad et al. [44]. A colony of *B. velezensis* CE 100 was grown in TSB medium overnight. Then, 10 µL of the bacterial culture broth was inoculated into 10 mL peptone broth followed by incubation at 30 °C for 2 days in a shaking incubator at 130 rpm. After the incubation period, the culture broth was added to 0.5 mL of Nessler’s reagent. The changing from a faint yellow to a dark brown color indicated ammonium production by *B. velezensis* CE 100.

Quantitative phosphate solubilization activity of *B. velezensis* CE 100 was determined as previously described [52] by measuring soluble phosphate in PVK broth amended with 10 g/L tricalcium phosphate (Ca_3_(PO_4_)_2_). A colony of *B. velezensis* CE 100 was grown in TSB medium for 3 days at 30 °C in a shaking incubator set at 130 rpm. Then, 2 mL of the bacterial culture broth (10^7^ CFU/mL) was inoculated into PVK medium (pH 7) and cultured under the same condition for 9 days. On 1, 3, 5, 7, and 9 days, 10 mL of bacterial culture broth was sampled and centrifuged at 10,000 rpm for 10 min. The supernatant was filtered through Whatman No. 6 filter paper, and 1 mL of filtrate was mixed with 2.5 mL of Barton’s reagent. The volume was adjusted to 50 mL with distilled water. After 10 min of incubation at room temperature, the intensity of the color development was measured on a UV spectrophotometer at 430 nm, and the amount of P solubilized was extrapolated from the standard curve. The pH variations of PVK medium during the growth of *B. velezensis* CE 100 were recorded.

### 4.6. Field Experiment Conditions

The field experiment was composed of the following three different treatment groups: (1) control (without fungicide or bacteria inoculation), (2) conventional treatment (fungicide), and (3) *B. velezensis* CE 100 inoculation (Figure 1C). Each treatment consisted of 15 trees planted at a spacing of 80 cm between lines and 180 cm within lines. A total area of about 121 m^2^ (11 m wide × 11 m long) divided into three blocks with a 50 cm buffer zone between the blocks was used in this experiment (Figure 1B). For each treatment, 2-year-old grafted seedlings (*Juglans mandshurica* Maxim. was used as the rootstock and *Juglans regia* L. as the scion) were planted in March 2019. The field was covered with a black weed barrier fabric to prevent weed growth. All trees were maintained under uniform field conditions. The treatments were applied on walnut trees from April to September 2020. At the start of the treatment period, the trees were approximately 100 cm high, with an average root collar diameter of 25 mm.

For the field treatment, *B. velezensis* CE 100 was grown in PB culture medium. The bacterial culture was prepared by spreading 100 µL of *B. velezensis* CE 100 culture broth (10^7^ CFU/mL) on 150 mm diameter petri dishes and incubated at 30 °C for 3 days. Then, two *B. velezensis* CE 100 culture plates were used to inoculate in PB medium (30 L), and the bacterial culture broth for field application was cultivated at 50 °C for 7 days.

The cell culture broth of *B. velezensis* CE 100 (10^5^ CFU/mL) was applied at a rate of 0.5 L per plant by drenching on the surface of the root zone. In addition, 1 L of diluted bacteria cell culture (1:1 ratio with water) was sprayed onto the leaves of the walnut trees. For conventional treatment, fluazinam fungicide (ISK Biosciences, Seoul, Korea) was used to spray on the leaves at a rate of 1.1 g/1000 m^2^, according to the manufacturer’s guidelines. For the control group, trees were sprayed with an equal volume of water to the volume used in the bacterial treatment. All treatments were applied twice per month from April to September 2020 during the experimental period.

### 4.7. Evaluation of the Biocontrol Activity and Plant Growth Promotion Potential of Bacillus velezensis CE 100 in Field Conditions 

In the 1st week of October 2020, the leaves of the walnut trees were examined for symptoms of anthracnose infection and disease severity (percent of infected plant area) based on symptoms of necrosis. The severity score was carried out on a scale of 0 to 4 based on the visual scale of necrosis on leaves using the modified method of Chiang et al. [53], where 0 = no signs of visible necrosis, 1 = necrotic lesions covering less than 25% of the total leaf surface, 2 = necrotic lesions covering 25–50% of the total leaf surface, 3 = necrotic lesions covering 50–75% of the total leaf surface, and 4 = necrotic lesions covering 75–100% of the total leaf surface. Severity was estimated using the midpoint of the value in the rating class for each plant. A total of nine trees were used to analyze the disease severity per treatment. The modified disease severity index (DSImodified) determined disease severity (%) using the following equation: DSI _(modified)_ (%) = [Mid_Q_ + (sum (class frequency × score of rating class))/(total number of plants × maximal disease index) × (Mid_Q+1_ − Mid_Q_)] × 100, where Mid_Q_ and Mid_Q+1_ are midpoint values of the Qth and (Q+1)th rating classes, respectively.

At the end of September 2020, the chlorophyll content was determined from all trees in each treatment. Twenty fully grown leaves were randomly selected from all sides, at the mid-height of each tree, and the chlorophyll content was measured using a chlorophyll meter SPAD-502 Plus (Konica Minolta, Tokyo, Japan).

Plant growth parameters, including shoot length, root collar diameter, and biomass, were measured from all the trees in October 2020. The biomass was measured after drying at 60–65 °C for 3 days.

### 4.8. Nutrient Content in Trees

Nine walnut trees were randomly selected to determine the total nitrogen and total phosphorus content in walnut trees from each treatment. The dried tree samples were pulverized and sieved through a 30 µm mesh screen. Total nitrogen was then analyzed using an elemental analyzer (Variomax CN Analyzer, Elementar Analysensysteme GmbH, Langenselbold, Germany) with a thermal conductivity detector (TCD) after combustion at 1200 °C with nitrogen and helium gas. The total phosphorus concentration in walnut trees was analyzed after digesting samples in nitric acid in a microwave oven (MARS Xpress, CEM Corporation, Matthews, NC, USA). The total phosphorus content in the plant samples was then measured using inductively coupled plasma optical emission spectrometry (ICP-OES) (Optima 8300, PerkinElmer, Waltham, MA, USA). The total nutrient content in walnut plants was calculated with the following formula: nutrient content (g/plant) = (dry weight (g/plant) × nutrient concentration (%/plant))/100 [32].

### 4.9. Data Analysis

The statistical analysis was performed using statistical analysis software (SAS) version 9.4 software (SAS Institute, Cary, NC, USA). Means were compared using the least significant difference (LSD) test of analysis of variance (ANOVA) at *p* = 0.05. The results are reported as mean ± standard deviation. ANOVA Table (Table S1) for all the data provide in this manuscript is provide in the supplementary material.

## 5. Conclusions

The findings of this study provided evidence that *B. velezensis* CE 100 could produce antifungal lytic enzymes, such as chitinase, protease, and β-1,3-glucanase, that inhibited the spore germination and mycelial growth of *C. gloeosporioides*, the phytopathogenic fungi that cause walnut anthracnose. The field application of *B. velezensis* CE 100 culture broth on walnut trees significantly reduced anthracnose severity in the orchard compared to the control. In addition, this study provides evidence that *B. velezensis* CE 100 can directly promote walnut growth via IAA auxin production, which increases root hair and lateral root growth for enhanced nutrient uptake. Moreover, *B. velezensis* CE 100 exhibited the potential to increase the availability of essential nutrients, such as nitrogen and phosphorus, via ammonium production and phosphate-solubilizing activity, respectively. Walnut trees inoculated with *B. velezensis* CE 100 showed increased nutrient uptake (total nitrogen and total phosphorus). In addition, inoculation with *B. velezensis* CE 100 increased the chlorophyll content, which consequently increases the photosynthetic rate, leading to an improved growth and biomass production of walnut trees. Therefore, *B. velezensis* CE 100 could potentially be applied as a PGPB in walnut production not only as a biocontrol agent against anthracnose disease but also as a biofertilizer to enhance tree growth and development.

## Figures and Tables

**Table 1:** Average chlorophyll content, shoot length, root collar diameter, and biomass production of walnut trees under different treatments.

Treatments	Chlorophyll Content (SPAD units)	Shoot Length (cm)	Root Collar Diameter (mm)	Biomass (g)
Control	33.2 ± 2.8 ^c^	184.6 ± 21.5 ^b^	44.1 ± 7.0 ^b^	613.5 ± 119.0 ^c^
Conventional	39.0 ± 2.3 ^b^	187.0 ± 22.5 ^b^	42.4 ± 9.4 ^b^	795.5 ± 101.6 ^b^
*B. velezensis* CE 100	44.6 ± 1.7 ^a^	227.6 ± 41.3 ^a^	57.2 ± 8.4 ^a^	1193.3 ± 200.5 ^a^

Means in a column followed by different superscripts are significantly different (*p* < 0.001).

**Table 2:** Total nitrogen and total phosphorus contents of walnut trees under different treatments.

Treatments	Nutrient Concentration (%/Plant)		Nutrient Content (g/Plant)	
	Total Nitrogen	Total Phosphorus	Total Nitrogen	Total Phosphorus
Control	1.50 ± 0.08 ^a^	0.21 ± 0.04 ^a^	9.85 ± 0.95 ^b^	1.37 ± 0.25 ^b^
Conventional	1.43 ± 0.06 ^a^	0.17 ± 0.02 ^b^	13.10 ± 2.05 ^a^	1.52 ± 0.28 ^b^
*B. velezensis* CE 100	1.28 ± 0.13 ^b^	0.19 ± 0.02 ^ab^	14.68 ± 1.86 ^a^	2.22 ± 0.57 ^a^

Means in a column followed by different superscripts are significantly different (*p* < 0.001).

## Data Availability

Data available on request from the corresponding author.

## Supplementary Materials

The following are available online at https://www.mdpi.com/article/10.3390/ijms221910438/s1.
