# Supplementary material for: Antifungal Activity of Bacillus velezensis CE 100 against Anthracnose Disease (Colletotrichum gloeosporioides) and Growth Promotion of Walnut (Juglans regia L.) Trees
Source: Int J Mol Sci. 2021 Sep 28;22(19):10438. doi: 10.3390/ijms221910438 (PMC8508943; doi:10.3390/ijms221910438)
Supplement: Supplementary file 1 [file ijms-22-10438-s001.zip › ijms-1370308-supplementary.pdf]

Table S1. ANOVA Table summary (cell growth, lytic enzyme production, antifungal activity against *Colletotrichum gloeosporioides* and plant growth promotion properties of *Bacillus velezensis* CE 100).

Parameters	Factor variable	Sum of squares	DF	Mean square	Critical value of t	F-ratio	*p*-value	LSD vales
Cell growth and lytic enzyme production of *B. velezensis* CE 100	[2] CFU (CFU)/mL	3269.7515	4.0000	817.4379	2.2281	55.2300	<0.0001	6.9990
	[3] Chitinase (units/mL)	3987.0734	4.0000	996.7684	2.2281	3161.8700	<0.0001	1.0215
	[3] β-1,3-Glucanase (units/mL)	2.8722	4.0000	0.7181	2.2281	226.2800	<0.0001	0.1025
	[3] Protease (units/mL)	27.6897	4.0000	6.9224	2.2281	5.6900	0.0118	2.0065
	[3] Crude enzyme (units/mL)	5512.1207	2.0000	2756.0603	2.4469	4403.3600	<0.0001	1.5806
Antifungal activity against *C. gloeosporioides*	[4] Spore germination (%)	1825.5256	2.0000	912.7628	2.4469	141.2800	<0.0001	5.0782
	[5] Mycelia growth inhibition (%)	429.0048	2.0000	214.5024	2.4469	17.8800	0.003	6.9195
	[6] Disease severity (%)	11282.5089	2.0000	5641.2544	2.0345	128.9000	<0.0001	5.4948
Plant growth promotion activity of *B. velezensis* CE 100 against on walnut trees	[7] IAA (µg/mL)	1.1560	4.0000	0.2890	2.2281	55.0600	<0.0001	0.1318
	[8] Phosphate solubilization(µg/mL)	1.5735	4.0000	0.3934	2.2281	28.8700	<0.0001	0.2124
	[8] pH	2.7090	4.0000	0.6772	2.2281	7.4600	0.0047	0.5483
	[a] Chlorophyll content (SPAD unit)	920.0519	2.0000	460.0260	2.0227	86.2200	<0.0001	1.7659
	[a] Shoot length (cm)	16331.6591	2.0000	8165.8295	2.0227	9.1600	0.0005	22.8210
	[a] Root collar diameter (mm)	1827.5119	2.0000	913.7560	2.0227	13.2100	<0.0001	6.3590
	[a] Biomass (g)	1582513.4710	2.0000	791256.7360	2.0639	36.7000	<0.0001	142.8500
	[b] Nitrogen concentration (%)	2.0204	2.0000	1.0102	2.0639	15.8900	<0.0001	0.2453
	[b] Phosphorous concentration (%)	0.0560	2.0000	0.0282	2.0639	6.9800	0.0041	0.0616
	[b] Nitrogen content (g/plant)	109.3234	2.0000	54.6617	2.0639	19.1100	<0.0001	1.6455
	[b] Phosphorous content (g/plant)	3.7422	2.0000	1.8711	2.0639	11.9800	0.0002	0.3844

Alpha (α) = 0.05. Superscripts; 2, 3, 4, 5, 6, 7 and, 8 indicate data presented in Figures 2, 3, 4, 5, 6, 7 and 8, respectively. Superscripts; a and b indicate data presented in Tables 1 and 2, respectively. DF is degrees of freedom and LSD is least significant difference.
